# Lateral hypothalamus and eating: cell types, molecular identity, anatomy, temporal dynamics and functional roles

**DOI:** 10.1038/s12276-025-01451-y

**Published:** 2025-05-01

**Authors:** Deok-Hyeon Cheon, Sheejune Park, Jihyun Park, MinSeo Koo, Hyun-Hyung Kim, Seol Han, Hyung Jin Choi

**Affiliations:** 1 Department of Biomedical Sciences, Seoul National University College of Medicine, Seoul, Republic of Korea; 2 Department of Anatomy and Cell Biology, Seoul National University College of Medicine, Seoul, Republic of Korea; 3 Department of Brain and Cognitive Sciences, Seoul National University, Seoul, Republic of Korea; 4 Neuroscience Research Institute, Seoul National University College of Medicine, Seoul, Republic of Korea; 5 Wide River Institute of Immunology, Seoul National University, Gangwon-do, Republic of Korea

**Keywords:** Hypothalamus, Motivation, Reward

## Abstract

The lateral hypothalamus (LH) is a central hub orchestrating eating behavior through its complex cellular, anatomical and temporal organization. The LH is characterized by high heterogeneity and functional complexity, with many aspects still unexplored. Here we synthesize recent advances in understanding the role of the LH in eating regulation across multiple dimensions. At the cellular level, the LH contains diverse neuronal populations that contribute to distinct roles in behavior. Anatomically, we divided the LH into four regions—anteromedial, anterolateral, posteromedial and posterolateral—each with unique cellular compositions, circuit organizations and projection patterns. By integrating the temporal dynamics of each LH cell type during eating behavior, we identified how various LH cell types are involved in regulating the appetitive and consummatory phases of eating behavior. The LH also plays vital roles in associative learning and different types of eating behavior, including homeostatic, pleasure-induced and stress-induced eating. These insights into LH organization and function provide promising directions for therapeutic interventions in eating disorders and obesity, including drugs, deep brain stimulation and gene therapy.

## Lateral hypothalamus revealed as key eating behavior hub

The lateral hypothalamus (LH) is a key brain area involved in behaviors such as eating and drinking. This Review assesses how the LH controls eating, focusing on its complex organization. Researchers explore how different neurons in the LH work together to regulate eating. The study uses advanced techniques, including single-cell RNA sequencing, to identify various neuron types in the LH. These neurons have different roles in eating, with some promoting and others suppressing food intake. They mapped these neurons’ locations and connections within the LH, revealing four distinct regions. The findings show that LH neurons are active during different phases of eating: the appetitive phase and the consummatory phase. This research helps us understand how the brain controls eating behavior, which could lead to new treatments for eating disorders.

This summary was initially drafted using artificial intelligence, then revised and fact-checked by the author.

## Introduction

The lateral hypothalamus (LH) has long been recognized as a critical brain region involved in diverse behaviors including eating, drinking, thermoregulation and energy expenditure. This Review specifically focuses on eating behavior, as it represents one of the most extensively studied and fundamentally important functions of the LH. While traditionally regarded as an ‘eating center’, recent advances in neuroscience techniques have revealed unprecedented complexity in its organization across cellular diversity, anatomical structure and temporal dynamics.

The LH exhibits sophisticated organization across multiple levels. At the cellular level, it contains diverse neuronal populations with distinct functions in eating regulation. GABAergic neurons promote eating^1–4^, glutamatergic neurons typically suppress it^5–7^, and specialized populations expressing leptin receptor (Lepr), neurotensin (Nts) and orexin (Orx) modulate specific aspects of eating behavior. This cellular diversity is intimately linked to anatomical organization, with different neuronal subtypes and circuits distributed across distinct regions of the LH.

To understand how these diverse neuronal populations work together, we examined their spatial organization within the LH. Specifically, we analyzed the distribution of cell types and circuits along the anteroposterior and mediolateral axes, identifying four distinct regions: anterolateral, anteromedial, posterolateral and posteromedial. Each region has characteristic cellular compositions and circuit organizations. However, these boundaries are not absolute; substantial overlap in cell-type distributions underscores the interconnected nature of LH circuits.

The temporal dynamics of LH circuits are critical for regulating eating behavior, with different neuronal populations exhibiting distinct activity patterns during the appetitive and consummatory phases of eating^3,6,8–14^. While appetitive and consummatory behaviors exhibit unique characteristics, their underlying neural mechanisms remain elusive. Recent advances in in vivo imaging and optogenetic techniques have enabled precise investigations into the roles of specific LH subpopulations. Interestingly, the LH appears to regulate both the appetitive and consummatory phases of eating behavior by engaging distinct neuronal populations and mechanisms tailored to each phase, ensuring precise coordination of eating behavior.

In this Review, we explore the organization and function of the LH in eating behavior by examining its molecularly distinct cell types, their anatomical distribution and their dynamic activity patterns. Our goal is to develop a comprehensive framework that integrates these cellular, anatomical and temporal dimensions. By organizing the LH’s neural populations into four anatomically distinct regions and analyzing how they operate during different phases of eating, we synthesized recent advances in LH research while highlighting important questions that remain unanswered.

## Cellular types and molecular identity in LH

### GABAergic and glutamatergic neurons

**Fig. 1:**
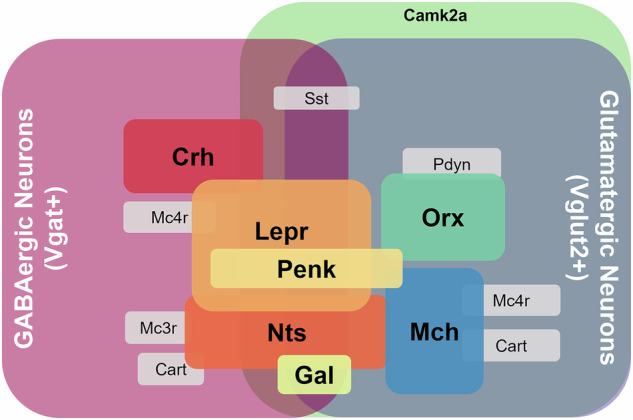
Molecular characterization of neuronal subtypes in the LH. The diagram depicts the complex organization of distinct neuronal populations defined by their molecular markers. GABAergic neurons and glutamatergic neurons are shown in pink and gray backgrounds respectively. Camk2a neurons are indicated in green. Within these broader populations, several specialized neuronal subtypes are identified, including neurons expressing Crh, Lepr, Nts, Orx and Mch. Additional neuronal markers, including Penk, Gal, Sst, Pdyn, melanocortin receptors (Mc3r/Mc4r) and Cart, are also represented. The overlapping regions between these populations illustrate the coexpression patterns of these molecular markers.

Recent advances in single-cell RNA sequencing (scRNA-seq), fluorescence in situ hybridization (FISH), immunohistochemistry (IHC) and transgenic reporter mouse models have enabled comprehensive molecular profiling of LH neurons. The LH contains multiple molecularly distinct neuronal populations (Fig. 1) that serve diverse functions. Recent studies have revealed that these populations can be categorized primarily on the basis of their neurotransmitter identity and neuropeptide expression patterns^15,16^. The two predominant neuronal populations are defined by their inhibitory or excitatory properties. The major inhibitory population consists of GABAergic neurons that express the vesicular GABA transporter (Vgat (Slc32a1)) (IHC and Tg mouse)^3,5,7^, while the principal excitatory population is marked by expression of vesicular glutamate transporter 2 (Vglut2 (Slc17a6)) (scRNA-seq, FISH, IHC and Tg mouse)^5–7,17^. Although these populations were initially thought to be entirely distinct, contemporary research using expansion-assisted iterative FISH has demonstrated that some neurons in the LH can coexpress both Vgat and Vglut2, indicating a more complex organization of neurotransmitter systems in this region^16^. This finding challenges the traditional view of strictly separated inhibitory and excitatory circuits and suggests potentially more sophisticated signaling mechanisms.

Nts neurons exhibit a mixed neurotransmitter profile, with approximately 80% expressing Vgat and 20% expressing Vglut2 (FISH, IHC, RNA-seq and Tg mouse)^18^. These neurons show varying levels of coexpression with multiple molecular markers, including glucagon-like peptide 1 receptor (Glp1r), somatostatin (Sst), cocaine and amphetamine regulated transcript peptide (Cart) and melanocortin 3 receptor (Mc3r)^18^. Notably, about 95% of Nts neurons coexpress galanin (Gal) (Tg mouse, FISH and IHC)^19^, and they show markedly overlap with melanocortin 4 receptor (Mc4r) neurons in the LH, with about 75% of Mc4r neurons also coexpressing Nts (Tg mouse and IHC)^20^.

Proenkephalin (Penk) neurons represent a complex population with mixed neurotransmitter identities. These neurons partially coexpress Vglut2 (52%) and Vgat (42%). Interestingly, they show minimal overlap with other major LH populations, with only 17% expressing Lepr. The majority (76%) of Penk neurons express Penk alone, while 17% coexpress Lepr, and less than 1% express all three markers (Tg mouse, IHC and FISH)^21^.

Corticotropin-releasing hormone (Crh) neurons predominantly exhibit a GABAergic phenotype, with about 82% expressing Vgat and only 10% expressing Vglut2 (RNAscope and IHC)^22^.

Lepr neurons demonstrate notable overlap with other populations, particularly Crh neurons, as approximately 50% of Crh neurons coexpress Lepr and 52% of Lepr neurons coexpress Crh^22^. While the majority of Lepr neurons are Vgat positive (Tg mouse and scRNA-seq)^10,14^, studies have also reported a Vgat-negative population, approximately 40% (Tg mouse, scRNA-seq and RNAscope)^12^. In addition, MC4R neurons show roubst leptin responsiveness, with approximately 80% demonstrating colocalization with pSTAT3 after leptin administration^20^.

Orx neurons predominantly express Vglut2 or Vglut1 (scRNA-seq, FISH, IHC and RNAscope)^15,23^. They also display extensive coexpression patterns with several neuropeptides. The majority of these neurons coexpress the endogenous opioid peptides prodynorphin (Pdyn) and Penk (scRNA-seq, FISH and IHC)^15,24^, showing nearly complete overlap with dynorphin (IHC and FISH)^24,25^ and islet amyloid polypeptide (IHC)^26^. They also demonstrate colocalization with Nts (FISH and IHC)^27^.

Melanin-concentrating hormone (Mch) neurons predominantly express Vglut1 and Vglut2, although some express glutamic acid decarboxylase 67 (Gad67). Distinct from Orx neurons (scRNA-seq, FISH and Tg mouse)^15,28,29^, Mch neurons coexpress neuropeptides such as Cart and as well as receptors including the neuropeptide Y receptor 5 (Npy5r), Mc4r and Lepr^30,31^.

Gal neurons demonstrate notable coexpression patterns, with approximately 50% expressing Vgat (Tg mouse and IHC)^32^. Notably, these neurons overlap substantially with the Nts population, as nearly 95% of Nts neurons coexpress Gal^19^. FISH studies have revealed that Gal neurons exhibit simultaneous expression of both Vgat and Vglut2 (ref. ^16^).

Calcium/calmodulin-dependent protein kinase II α (Camk2a) neurons express Camk2a, a marker commonly used for excitatory neurons. Most Vglut2-expressing neurons in the LH express Camk2a; however, only about 64–79% of Camk2a-expressing neurons express Vglut2 (Tg mouse and IHC)^11,33^. Regarding the remaining fraction, there are mixed findings: some studies report that they express Vgat and Gad2 (ref. ^33^), while others suggest that they do not express either Vgat or Vglut2 (ref. ^11^).

This molecular heterogeneity underscores the functional complexity of the LH in regulating eating behavior. Each neuronal population, defined by its unique combination of molecular markers, contributes to distinct aspects of eating behavior regulation.

## Anatomical locations and projections in LH

Given the broad spatial extent of the LH and its diverse cellular heterogeneity, we propose subdividing the region along its anteroposterior and mediolateral axes. Due to the lack of clear important landmarks within the LH, we used a geographic approach that establishes boundaries based on numerical coordinates rather than distinct anatomical landmarks. This method provides a practical and systematic way to delineate its subregions and offers guidance for stereotaxic surgery. This approach categorizes the LH into four subdivisions: anteromedial (am), anterolateral (al), posteromedial (pm) and posterolateral (pl) regions. Based on stereotaxic coordinates provided in The Mouse Brain in Stereotaxic Coordinates^34^, the LH is described with anterior and posterior boundaries at approximately −0.8 mm and −2.2 mm relative to bregma, respectively. To provide a clear distinction between anterior and posterior regions, we use −1.5 mm as a midline reference. Similarly, given that the mediolateral extent of the LH generally falls between ±0.8 mm and ±1.2 mm, we use ±1.0 mm as a midline to distinguish the medial and lateral compartments.

**Fig. 2:**
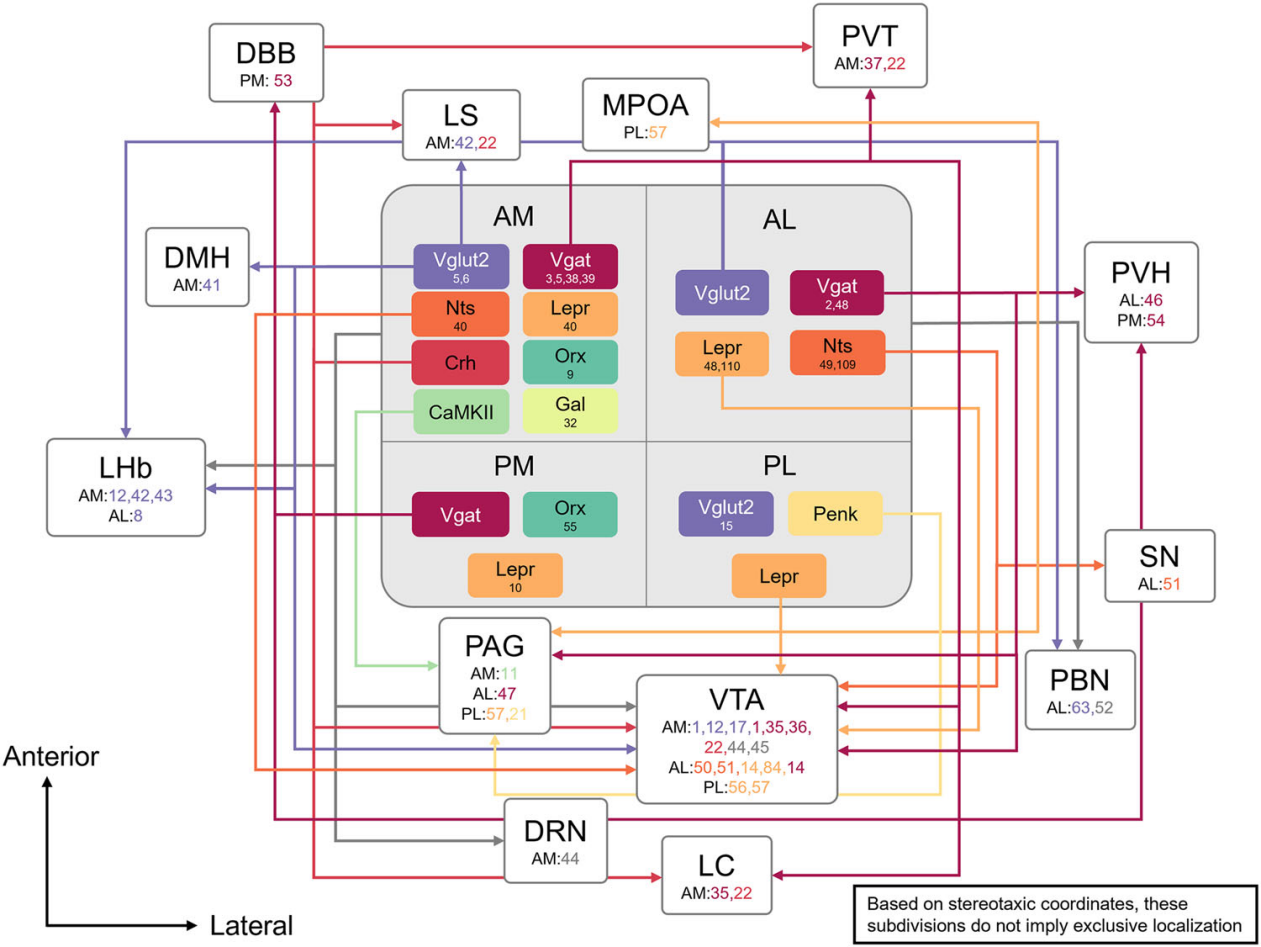
Anatomical organization of LH and neural circuits. The diagram illustrates the anatomical and molecular organization of hypothalamic circuits and their projections. The central region is divided into four subregions: anteromedial (AM), anterolateral (AL), posteriomedial (PM) and posteriolateral (PL) areas. Each region contains distinct neuronal populations characterized by specific molecular markers including Vglut2, Vgat, Nts, Lepr, Crh, Orx, Camk2a, Gal, Sst and Penk, with their relative abundance indicated by numbers. These hypothalamic neurons form extensive connections with multiple brain regions, including the DBB, LS, MPOA, PVT, DMH, LHb, PVH, PAG, VTA, SN, PBN, DRN and LC. Colored arrows represent distinct neural pathways, and numbers associated with each region and color represent reference numbers for the corresponding cell types in the literature. Note that these subdivisions are based on reported stereotaxic coordinates and do not imply exclusive localization.

Although various neuronal populations (for example, Vgat and Vglut2 neurons) are broadly distributed across the LH, in this Review we compile and synthesize established findings from previous studies to propose a coordinate-based subdivision that aligns with recognized anatomical boundaries. This framework offers a practical approach to improve targeting accuracy in experimental studies. It is important to clarify that our intent is not to suggest a restricted or exclusive distribution of these gene expressions; rather, we have organized reported experimental results that targeted specific LH regions based on the stereotaxic coordinates provided in the literature (Table 1 and Fig. 2).

**Table 1:** Neural circuit connectivity and control of eating behavior by cell-type-specific projections from LH subregions.

Area	Cell type	Projection (to)	Modulation	Experimental conditions	Eating behavior	Coordinate (AP, ML, DV)	Ref.
amLH	Vgat	LC	Activation	Optogenetics, free-feeding, chow	↑	−1.2, 1.0, −5.2	^35^
		PVT	–	X	X	−1.3, 0.9, −5.15	^37^
		VTA	Activation	1: optogenetics, free-feeding, sucrose 35: optogenetics, free-feeding, chow 36: optogenetics, free-feeding, chow	↑	1: −0.4 to −0.8, 1.0,−4.9 to −5.2 35: −1.2, 1.0, −5.2 34: −1.3, 1.0, −5.2	^1,35,36^
		X	Activation	3: optogenetics, chemogenetics, free-feeding, chow 5: optogenetics, free-feeding, chow 38: optogenetics, free-feeding, sucrose 39: chemogenetics, context induced feeding, chow	↑	3: −1.3, 0.9, −4.85 5: −1.23, 1.0, −5.15 38: −1.25, 1.0, −4.9 39: −1.2, 1.0, −5.2	^3,5,38,39^
	Nts	X	Activation	Chemogenetics, free-feeding, chow	↑	−1.3, 0.9, −5.2	^40^
	Crh	VTA	Activation	Optogenetics, free-feeding, chow	↑	−1.34, 0.95, −4.95	^22^
		LC	Activation	Optogenetics, free-feeding, chow	↑	−1.34, 0.95, −4.95	^22^
		LS	–	X	X	−1.34, 0.95, −4.95	^22^
		PVT	–	X	X	−1.34, 0.95, −4.95	^22^
	Gal	X	Activation	Chemogenetics, progressive ratio test, sucrose	↑	−1.35, 0.9, −5.4	^32^
	Lepr	X	Activation	Optogenetics, free-feeding, chow	↓	−1.3, 0.9, −5.2	^40^
	Vglut2	DMH	Activation	Optogenetics, chow	↓	−1.3, 0.9, −5.3	^41^
		LHb	Inhibition	Chemogenetics, taste preference test, sucrose and denatonium	↑	−1.34, 1, −5.2	^42^
			Inhibition	Optogenetics, ensure	↑	−1.0, 0.9, −6.0	^43^
			–	X	X	−1.35, 0.95, −5.15	^12^
		LS	Inhibition	Chemogenetics, taste preference test, sucrose and denatonium	↑	−1.34, 1.0, −5.2	^42^
		VTA	Activation	Optogenetics, free-feeding, chow and chocolate pellets	↓	−1.3, 1.0, −5.2	^17^
			–	X	X	−1.35, 0.95, −5.15	^12^
		X	Acitvation	Optogenetics, free-feeding, chow	↓	−1.23, 1.0, −5.15	^5^
			–	X	X	−1.3, 0.9–1.0, −5.1	^6^
	CaMKII	PAG	Activation	Optogenetics, free-feeding, chow	↑	−1.3, 1.0, −5.0	^11^
	Orx	X	Ablation	Ablation, foods of diverse tastes and textures (chow, peanut butter, yogurt, strawberry milkshake, sucralose solution)	↑	−1.38, 0.95, −5	^9^
	Non specific	DRN	Activation	Optogenetics, sucrose	↑	−1.2, 1.0, −5.2	^44^
		LHb	Activation	Optogenetics, sucrose	↓	−1.2, 1.0, −5.2	^44^
		VTA	Activation	Optogenetics, sucrose	↑	−1.2, 1.0, −5.2	^44^
			–	X	X	−0.4, 1.0, −4.9	^45^
alLH	Vgat	PAG	Activation	Optogenetics, free-feeding, chow	↓	−1.4, 1.1, −4.9	^47^
		PVH	Activation	Optogenetics, free-feeding, chow	↑	−1.0, 1.1–1.3, −5.0	^46^
		VTA	Activation	Optogenetics, free-feeding, chow	↑	−0.9 to −1.5, 1.1, −4.75 to −5.2	^14^
		X	Activation	2: chemogenetics, free-feeding, entailing calories (food, sucrose and ethanol), lacking calories (saccharin and water) and those lacking biological relevance (wood) R48: chemogenetics, free-feeding, chow	↑	2: −1.2, 1.2, −5.1 48: −1.2, 2.0, −5.4	^2,48^
	Nts	VTA	–	X	X	−1.34, −1.13, −5.2	^50,51^
		SN	–	X	X	−1.34, −1.13, −5.2	^51^
		X	Activation	Chemogenetics, free-feeding, chow	↓	−1.34, 1.05, −5.2	^49^
	Lepr	VTA	Activation	Optogenetics, free-feeding, chow	↔	−0.9 to −1.5, 1.1, −4.75 to −5.2	^14^
			–	X	X	−1.34, 1.13, −5.2	^84^
		X	Activation	Chemogenetics, free-feeding, chow	↓	−1.2, 2, −5.4	^48^
			–	X	X	−1.1, 1.9, −5.3	^110^
	Vglut2	LHb	Activation	Optogenetics, aversive response	↓	−1.1,1.1,−4.5	^8^
	Nonspecific	PBN	–	X	X	−1.2, 1.2, −5.2	^52^
pmLH	Vgat	DBB	Activation	Optogenetics, free-feeding, chow	↑	−1.5, 0.9, −5.1	^53^
		PVH	Activation	Optogenetics, free-feeding, chow	↑	−1.6, 1.0, −5.1	^54^
	Lepr	X	Activation	Optogenetics, free-feeding, chocolate-flavored snack, sucrose gel	↑	−1.5, 0.9, −5.25	^10^
	Orx	X	Activation	Pharmacological test	↑	−1.5, 0.7, −5.0	^55^
plLH	Lepr	VTA	Activation	Optogenetics, progressive ratio test, chow	↑	−1.55, 1.1, −5.2	^56^
			Inhibition	Optogenetics, chemogenetics, high-fat diet	↔	−1.65, 1.12, −5.25	^57^
		PAG	Activation	Optogenetics, chemogenetics, high-fat diet	↑	−1.65, 1.12, −5.25	^57^
		MPOA	Activation	Chemogenetics, high-fat diet	↔	−1.65, 1.12, −5.25	^57^
	Penk	PAG	Activation	Chemogenetics, free-feeding, high-fat diet	↑	−1.65, 1.12, −5.25	^21^

This table summarizes neural circuit manipulations and their effects on eating behavior across different LH subregions. The ‘Area’ column indicates anatomical subregions of the LH, including anterior medial (amLH), anterior lateral (alLH), posterior medial (pmLH) and posterior lateral (plLH) regions. ‘Cell type’ refers to genetic markers, such as Vgat, Vglut2, Nts, Crh, Gal, Lepr, Orx, Sst and Penk. ‘Projection (to)’ specifies target brain regions, including LC, PVT, VTA, DMH, LHb, LS, PAG, DRN, PVH, PBN, DBB, MPOA and SN. When marked with ‘X’ in this column, it indicates that circuit mapping was not performed. The ‘Modulation’ column indicates the type of neural manipulation performed: activation, inhibition, ablation or identified connection without specific manipulation (–). ‘Coordinate (AP, ML, DV)’ provides stereotaxic coordinates used for targeting specific LH subregions, where AP (anteroposterior), ML (mediolateral) and DV (dorsoventral) define spatial positioning relative to the bregma. ‘Eating behavior’ shows the effect on eating behavior, where upward arrows (↑) indicate increased eating behavior, downward arrows (↓) indicate decreased eating behavior, horizontal arrows (↔) indicate no effect on eating behavior, and ‘X’ indicates that eating behavior was not investigated. ‘Ref.’ provides reference numbers from the cited literature. Note that these subdivisions are based on reported stereotaxic coordinates and do not imply exclusive localization.

### Anteromedial LH (amLH)

The amLH region contains diverse neuronal populations influencing eating behavior through distinct projection patterns. Vgat neurons project to the locus coeruleus (LC), paraventricular thalamus (PVT) and ventral tegmental area (VTA). Activation of LC-projecting and VTA-projecting Vgat neurons increases eating behavior^1,35,36^, while the effect of PVT projections remains to be further characterized^37^. Although the precise projection targets were not examined in these studies, activation of Vgat neurons in the amLH has also been shown to stimulate eating behavior^3,5,38,39^. Nts neurons in amLH increase eating behavior^40^. Crh neurons project to multiple regions, including the VTA, LC, lateral septum (LS) and PVT. The activation of VTA and LC projections shows increased eating behavior, while LS and PVT effects remain uncharacterized^22^. Activation of Gal neurons increases eating behavior^32^, whereas activation of Lepr neurons decreases it^40^; specific projection targets are yet to be identified. Vglut2 neurons in this region exhibit multifaceted effects on eating behavior through their diverse projection targets. Dorsomedial hypothalamus (DMH)-projecting Vglut2 neurons decrease eating when activated^41^, while lateral habenula (LHb)-projecting neurons show an increase in eating behavior when inhibited^42,43^. LS-projecting neurons increase eating through inhibitory mechanisms, and VTA-projecting neurons decrease eating when activated^1,17,42^. Camk2a neurons that project to the periaqueductal gray (PAG) increase eating behavior upon activation^11^. Orx neurons contribute to eating regulation, as their ablation leads to increased eating behavior^9^. Studies without cell type specificity show activation of LHb projections decreases eating behavior, while activation of dorsal raphe nucleus (DRN) and VTA projections increases eating behavior^44,45^.

### Anterolateral LH (alLH)

The alLH region comprises several defined neuronal populations with specific effects. Vgat neurons project to multiple regions, including the PAG, VTA and paraventricular hypothalamus (PVH). Activation of these neurons increases eating behavior regardless of their projection targets^14,46–49^. Nts neurons project to the VTA and substantia nigra (SN); however, their effects remain under investigation^50,51^. Similarly, VTA-projecting Lepr neurons also have unclear regulatory effects on eating behavior^14^. Activation of other projections also reduces eating^48^. Vglut2 neurons project to the LHb and parabrachial nucleus (PBN), with both suppressing eating behavior^8^. Without distinguishing specific cell types, projections to the PBN are observed; however, the precise impact of these generalized projections on eating behavior has yet to be fully elucidated^52^.

### Posteromedial LH (pmLH)

The pmLH region includes defined neuronal populations with varying effects on eating behavior. Lepr neurons in pmLH promote seeking or consummatory behaviors^10^. Vgat neurons projecting to PVH and diagonal band of Broca (DBB) increase eating behavior when activated^53,54^. Orx neurons in this region also increase eating behavior upon activation, although their specific projection patterns have yet to be identified^55^.

#### Posterolateral LH (plLH)

The plLH region includes defined neuronal populations with varying effects on eating behavior. Lepr neurons project to regions such as the VTA, PAG and medial preoptic area (MPOA). Activation of VTA-projecting and PAG-projecting neurons increases eating behavior, while inhibition of VTA projections and activation of MPOA projections shows no notable effect on eating^56,57^. The region also contains Vglut2 neurons, although their effects on eating behavior require further investigation^15^. In addition, Penk neurons project to the PAG, with activation increasing eating behavior^21^.

### Upstream of LH

The LH receives diverse upstream inputs from multiple brain systems, with distinct subregions integrating specific regulatory signals (Table 2).

**Table 2:** Neural circuit connectivity and control of eating behavior by inputs to LH subregions.

Area	Cell type	Projection (from)	Modulation	Experimental conditions	Eating behavior	Coordinate (AP, ML, DV)	Ref.
amLH	Vgat	Septum	Activation	Optogenetics, chemogenetics, free-feeding, chow	↓	−1.3, 1.0, −5.0	^58^
	CaMKII	MPOA	Activation	Optogenetics, chemogenetics, free-feeding, food pellet	↑	−1.3, 1.0, −5.0	^11^
	Agrp	ARC	Activation	Optogenetics, chemogenetics, food pellets	↑	−1.3, 1.0, −4.7	^60^
	Nonspecific	mPFC	Activation	Optogenetics, chemogenetics, progressive ratio task, chow	↓	−1.3, 1.0, −4.9	^61^
	Glp1r	LS	Activation	Chemogenetics, free-feeding, fast–refeeding, chow	↓	−1.2, 1.0, −5.1	^59^
alLH	CamKII	IC	Activation	Optogenetics, chemogenetics, free-feeding, chow	↓	−1.28, 1.23, −5.28	^62^
	Vglut2	PBN	Activation	Optogenetics, operant fixed ratio, progressive ratio, fast–refeeding, chow, sucrose	↓	−1.1 to −1.3, 1.15, −4.9	^63^
	D1	NAc	Activation	65: optogenetics, lickometer, liquid fat or sucrose 66: optogenetics, chow, liquid fat, high-fat diet	↓	65: −1.2, 1.2, −4.75 66: −1.17, 1.17, −4.9	^65,66^
	Agrp	ARC	Activation	Optogenetics, free-feeding, operant progressive ratio, food-choice test chow, food pellets, nonnutritive gels	↑	−1.4, 1.2, −4.7	^64^
plLH	Dbh	LC	Activation	Optogenetics, free-feeding, chow, food pellets	↓	−1.7, 1.71, −4.81 to −4.86	^67^
	Nts	LS	Activation	Chemogenetics, optogenetics, post-stress free-feeding	↓	−1.8, 1.5, −5.25	^18^
pmLH	Vglut2	BNST	Activation	Optogenetics, food preference test, chow, high-fat diet	↑	−1.7, 0.9, −4.75	^7^

This table maps the neural circuit organization of inputs to LH subregions, detailing how different afferent populations connect to and influence LH neurons to regulate eating. The ‘Area’ column indicates anatomical subregions of the LH, including anterior medial (amLH), anterior lateral (alLH), posterior medial (pmLH) and posterior lateral (plLH) regions. Cell types are identified by genetic markers in the input regions, including Vgat neurons, Camk2a neurons, Agrp neurons, Glp1r neurons, D1r neurons, Dbh neurons and Nts neurons. These cells project from various brain regions including the septum, MPOA, ARC, LS, LC, IC and NAc. The ‘Modulation’ column indicates the type of neural manipulation performed. ‘Coordinate (AP, ML, DV)’ provides stereotaxic coordinates used for targeting specific LH subregions, where AP (anteroposterior), ML (mediolateral) and DV (dorsoventral) define the spatial positioning relative to the bregma. ‘Eating behavior’ shows the effect on eating behavior, where upward arrows (↑) indicate increased eating behavior, downward arrows (↓) indicate decreased eating behavior, horizontal arrows (↔) indicate no effect on eating behavior, and ‘X’ indicates that eating behavior was not investigated. ‘Ref.’ provides reference numbers from the cited literature. Note that these subdivisions are based on reported stereotaxic coordinates and do not imply exclusive localization.

The amLH receives inputs that either promote or suppress eating. The septum sends Vgat projections to amLH, reducing eating^58^, while the LS Glp1r neurons also contribute to eating suppression^59^. By contrast, MPOA CaMKII neurons promote eating through projections to amLH^11^, and the arcuate nucleus (ARC) enhances eating through its widespread connections across the LH^60^. In addition, the medial prefrontal cortex (mPFC) projects to the amLH and integrates control over eating behavior under stress, leading to reduced eating^61^.

The alLH receives several inputs linked to eating suppression. The insular cortex (IC) CaMKII neurons send projections to alLH, reducing eating^62^, while the PBN Vglut2 pathway is also associated with lower food intake^63^. By contrast, the ARC projects to the alLH and promotes eating behavior^64^. In addition, the nucleus accumbens (NAc) D1 neurons project to alLH and are implicated in reward-related modulation of eating behavior^65,66^, and the ARC increases eating behavior.

The plLH receives brainstem and limbic inputs that reduce eating. The LC dopamine β-hydroxylase (Dbh) neurons project to plLH and are linked to eating suppression^67^, while the LS Nts neurons also send projections to plLH, contributing to reduced eating^18^.

The pmLH receives input that increases eating. The BNST Vglut2 projections to pmLH are associated with increased eating^7^.

This organized upstream network allows precise regulation of eating behavior through multiple parallel pathways.

## Role of LH in the temporal dynamics of eating behavior phases

### Distinction between appetitive and consummatory behavior phases

How did the distinction between appetitive behavior and consummatory behavior arise? This differentiation stems from a fundamental question of what drives animal behavior and whether it is innate or the result of learning. Behaviorists argue that behaviors are variable and flexible, shaped primarily through learning^68^. By contrast, ethologists emphasize species-specific, stereotypical behaviors triggered by specific stimuli^69,70^. To reconcile these opposing perspectives, researchers have distinguished between appetitive and consummatory behavior within a behavior sequence, emphasizing their complementary roles in achieving and satisfying needs. Appetitive behaviors involve exploratory or goal-oriented actions leading up to the final behavior, while consummatory behaviors encompass actions that fulfill the goal and satisfy the individual’s needs^70–73^.

Eating behavior can be broadly divided into two phases: the appetitive phase, which occurs first, and the consummatory phase, which follows^74–76^. Within this framework, in the context of eating behavior, appetitive behaviors—such as foraging, predatory hunting and approaching food—involve exploring or moving toward a food source^73,74^. These behaviors are adaptive, highly variable and flexible, relying on learning from food cues and trial-and-error processes to optimize food acquisition. By contrast, consummatory behaviors occur after the goal has been contacted through appetitive behaviors^70,73,77^. In the context of eating behavior, consummatory behaviors consist of actions such as biting, chewing and swallowing, which are innate responses to stimuli and often display stereotypical patterns within a given species^73,74^.

In natural environments, animals must engage in appetitive behaviors such as foraging or hunting to secure food before energy deficits occur. Despite the importance of appetitive behavior, due to the challenges of separating the two phases, research has predominantly focused on consummatory behaviors, such as food intake. However, advances in behavioral experiments and in vivo imaging have made it possible to study appetitive behaviors independently. Notably, in the context of eating behavior, LH appears to regulate both phases, serving as a central hub in the eating sequence. The following chapter explores the LH’s role in appetitive and consummatory behaviors and the temporal dynamics of its activity.

### GABAergic neurons

The LH directly regulates appetitive and consummatory behaviors through its various subpopulations. Unlike agouti-related peptide (Agrp) neurons, which are rapidly inhibited by food-predicting cues, specific neurons in the LH remain active not only until food consumption begins but also throughout the consummatory phase, thereby promoting both appetitive and consummatory behaviors^41,78,79^. LH GABAergic neurons are among these. Optogenetic stimulation of LH GABAergic neurons increases food intake, time spent in food-paired areas and reward-seeking behaviors, highlighting their role in promoting appetitive and consummatory behaviors^3^. Conversely, optogenetic inhibition of these neurons robustly reduces food intake and reward-seeking behaviors, demonstrating their necessity for these functions^3^. Similarly, chemogenetic activation of these neurons induces indiscriminate gnawing-like consummatory behavior, regardless of the caloric value of the object^1,2^. Furthermore, we have shown that chemogenetic activation in nonhuman primates also increases appetitive behaviors, such as approaching the hand to food and enhancing operant tasks performed to obtain food^4^. One of the major projections of LH GABAergic neurons is to the VTA, where inhibitory inputs from LH GABAergic neurons to VTA GABAergic neurons increase dopamine release in the NAc, thereby enhancing appetitive behavior^1,80^. Dopamine receptor 1 (D1r) neurons in the NAc inhibit LH GABAergic neurons, with their activity decreasing during the consumption phase, highlighting a reciprocal relationship among VTA, NAc and LH GABAergic neurons in regulating the appetitive and consummatory phases^65^.

**Fig. 3:**
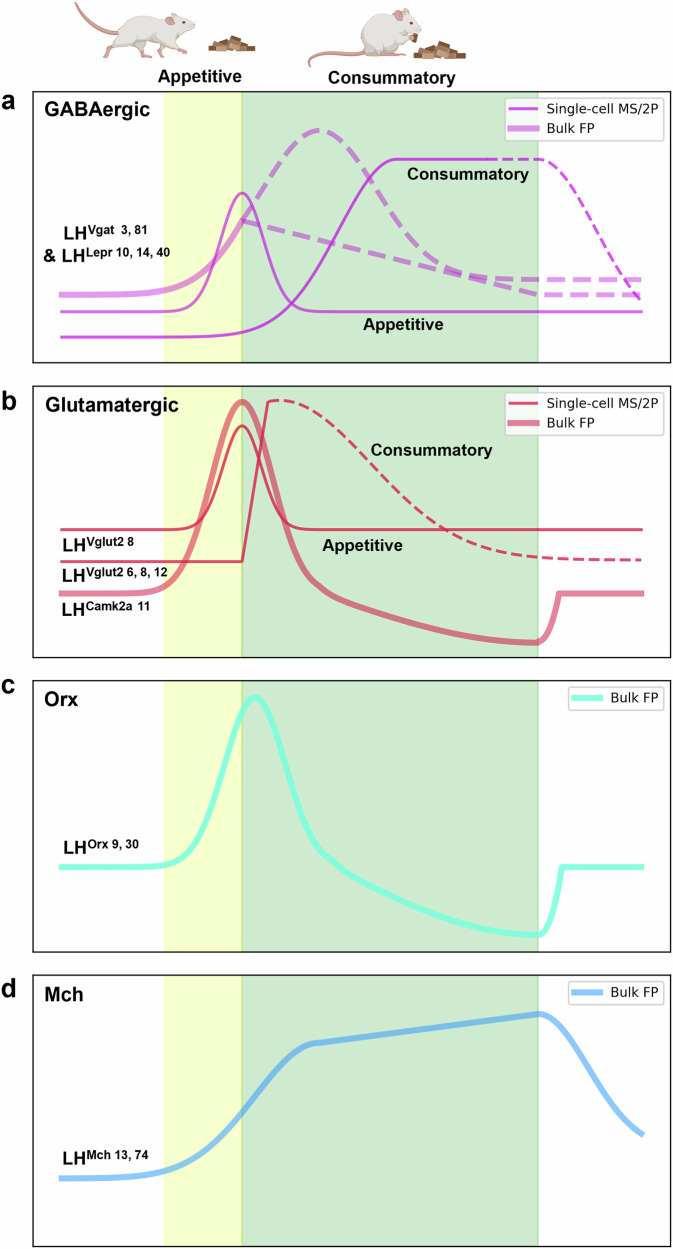
Temporal dynamics of distinct LH neuronal populations during appetitive and consummatory phases of eating behavior. This figure reconstructs the activity of five cell types in the LH during eating behavior based on calcium dynamics measured with genetically encoded calcium indicators as presented in the referenced studies. The *x* axis represents time, and the *y* axis represents calcium dynamics. The thick lines represent the calcium dynamics of bulk cells measured using fiber photometry, while the thin lines represent calcium dynamics measured at single-cell resolution using either a one-photon miniaturized microendoscope or a two-photon microscope. The dashed lines indicate activity that is not clearly defined. The yellow box indicates the appetitive phase, and the green box indicates the consummatory phase. The relative differences in size between the graphs are arbitrary and do not reflect actual differences in activity magnitude between them. **a**, Calcium dynamics of GABA and Lepr neurons during eating behavior. The appetitive population represents the neuronal population activated from the onset of appetitive behavior, while the consummatory population represents the neuronal population activated from the onset of consummatory behavior. **b**, Calcium dynamics of Glut and Camk2α neurons during eating behavior. The appetitive population represents the neuronal population activated from the onset of appetitive behavior, while the consummatory population represents the neuronal population activated from the onset of consummatory behavior. **c**, Calcium dynamics of Mch neurons during eating behavior. **d**, Calcium dynamics of Orx neurons during eating behavior.

Fiber photometry results show that LH GABAergic neurons are activated when mice approach food, with activity peaking upon food contact and maintaining their activity or slowly decreasing during the consummatory phase (Fig. 3a)^81^. Similar activity patterns of these neurons were observed in fed-state mice during hedonic eating of peanut butter and in a nonedible object^81^. Miniaturized fluorescence microscope imaging revealed distinct populations of LHGABA active during the appetitive and consummatory phases^3^. Optogenetic and chemogenetic activation increased both appetitive and consummatory behavior, and because distinct neurons respond in each phase, it is suggested that LH GABAergic neurons drive both appetitive and consummatory behavior through distinct populations of neurons. Unlike the appetitive population, the consummatory population becomes active at the point of food contact and displays sustained elevated activity throughout the consummatory behavior^3^. This indicates that consummatory neurons may be involved not only in the initiation of eating but also in its continuation.

### Lepr neurons

Leptin, produced by adipocytes, acts on Lepr to reduce motivation for food and food intake, thereby maintaining long-term energy balance^82,83^. Lepr is widely expressed in brain regions such as the ARC, DMH, ventromedial hypothalamus, LH and nucleus of the solitary tract, with distinct roles in regulating eating behavior and energy balance^74,83^. The majority of LH Lepr neurons are a subpopulation of LH GABAergic neurons, accounting for approximately 20% of LH GABAergic neurons^14,48,56^.

The role of LH Lepr neurons remains controversial. Some studies suggest that these neurons reduce eating behavior in response to leptin administration^84^, while others indicate that they increase appetitive behavior without affecting consummatory behavior^14^. Yet, we and other studies propose that LH Lepr neurons enhance both appetitive and consummatory behaviors^10,57^. We believe that the controversy on the role of Lepr neurons in eating behavior discussed in our Review stems from conflicting experimental schemes to test appetitive and consummatory behaviors. In our experiment, phase context-specific optogenetic manipulation revealed that Lepr neurons drive both appetitive and consummatory behavior, highlighting the importance of meticulous event design in studying cell types and brain circuits involved in specific phases of eating^10^.

Fiber photometry results indicate that LH Lepr neurons become activated during the appetitive phase and exhibit increased activity during the consummatory phase as well^10^. However, findings from miniaturized fluorescence microscopy show that distinct populations of neurons are activated during the appetitive and consummatory phases, respectively^10,14^ (Fig. 3a). These results indicate that, similar to GABAergic neurons, phase-specific neurons within LH Lepr neurons may drive the corresponding specific behavior phases^10^.

### Glutamatergic neurons

LH glutamatergic neurons are well known for their role in suppressing appetitive and consummatory behaviors. Optogenetic studies revealed that activation of glutamatergic neurons induces satiety-like states and suppresses appetitive behaviors such as food seeking, while their inhibition increases food intake^5–7^. In particular, excitatory inputs from LH glutamatergic neurons to LHb and VTA GABAergic neurons are aversive and suppress consummatory behaviors^8,12,17,43^. However, optogenetic activation of Camk2α-positive neurons in the LH, which serve as a marker of excitatory neurons, increases hunting, prey capture and food pellet consumption, which may be attributed to the presence of Camk2α neurons that do not express Vglut2 (ref. ^11^).

Calcium imaging has revealed that different types of LH glutamatergic neurons exhibit distinct activity patterns during various phases of eating behavior. LH glutamatergic neurons, like GABAergic neurons, include distinct populations that are activated during the appetitive phase and the consummatory phase, respectively. Neurons activated during the appetitive phase respond to operant tasks for obtaining food or visual cues of food, peaking in activity at the moment of food contact, followed by a decrease.^8,41^. Meanwhile, neurons activated during the consummatory phase exhibit a sharp increase that is time-locked to food contact. As previous studies have not clearly defined bout duration, it remains unclear how the activity of these neurons changes over a longer timescale in the consummatory phase^6,8,12^. However, these neurons seem to exhibit a slightly sharper increase in activity at the moment of food contact compared with GABAergic consummatory neurons and respond more strongly to aversive tastants than to palatable ones^3,8,12^. This suggests that these neurons are more likely to be involved in momentary actions, such as stopping food intake, triggered by food contact itself or the taste of food, rather than in long-term processes such as post-oral effects. Camk2α-positive neurons also show heightened activity during appetitive behaviors such as predatory hunting, but their activity rapidly returns to baseline upon the transition from hunting to consuming the prey, as demonstrated by fiber photometry results^11^ (Fig. 3b).

Rather than simply suppressing appetitive or consummatory behaviors, LH glutamatergic neurons probably initiate the termination phase of feeding. Effective regulation of eating behavior requires both acceleratory mechanisms that drive appetitive and consummatory behaviors as well as braking mechanisms that initiate the termination phase of eating^74,75^. LH glutamatergic neurons probably function as this brake as they are activated upon food contact and respond more strongly to aversive stimuli than to palatable tastants^6,12^. However, given the heterogeneous nature of LH glutamatergic neuronal populations, their precise role remains a subject of debate. Future research targeting specific subpopulations will be crucial in clarifying their distinct contributions to feeding regulation.

### Orx/hypocretin neurons

Orx/hypocretin-producing neurons are another subpopulation within the LH. Chemogenetic activation of LH Orx neurons and intracerebroventricular administration of orexin-A increases motivation to acquire palatable food and food intake^55,85^, probably due to the promotion of appetitive behavior by Orx. Interestingly, contrary to chemogenetic results, knockout of Orx neurons in adult mice leads to overeating and obesity, while overexpression of Orx prevents diet-induced obesity^9,86^. These findings suggest that Orx neurons play an important role in regulating eating behavior and maintaining energy balance in a complex and context-dependent manner.

Orx neurons exhibit high activity in food-deprived states, are inhibited by glucose and leptin, and are activated by ghrelin^87–89^. On a shorter timescale during eating behavior, Orx neurons are activated by food-predicting cues and exhibit increased activity during appetitive behavior, suggesting that they are activated when the animal anticipates food^9,90^. Notably, their activity is not limited to responding to food cues but is sustained throughout the appetitive behavior until the onset of consummatory behavior, indicating that Orx neurons may actively promote appetitive behavior^91^. However, their activity rapidly decreases as soon as food consumption begins (even before post-ingestive effects occur), remains low throughout the consummatory phase and returns to baseline once eating is completed^9,89^ (Fig. 3c). Orx neurons, well known for inducing arousal^27,87^, are activated during the appetitive phase of eating behavior but show reduced activity upon food contact, suggesting that they may be more closely associated with foraging and food approaching than with consummatory behavior^89^.

### Mch neurons

Mch, another orexigenic subpopulation within the LH, also contributes to the regulation of eating behavior. Mch administration increases food intake, and Mch overexpression has been linked to increased adiposity and obesity^92,93^. In both normal mice and sweet-blind Trpm5-knockout mice, optogenetic activation of LH Mch neurons during the consumption of sucralose enhanced the preference for nonnutritive sucralose to levels comparable to sucrose^94^. In addition, optogenetic stimulation of Mch neurons during consumption has been shown to increase food intake by prolonging eating episodes. However, this stimulation alone was insufficient to trigger the initiation of eating, indicating that Mch neuron activation specifically sustains ongoing consumption rather than initiating it. These findings suggest that Mch neurons promote the consumption of specific foods by amplifying their nutritional value through post-oral mechanisms^74,94,95^. Recent studies have shown that chemogenetic activation of Mch neurons enhances food-seeking behavior in response to food cues, increasing both the speed and frequency of food-seeking actions^13^. Therefore, Mch neurons are thought to play a pivotal role in both motivating food acquisition and prolonging consummatory behavior.

Unlike Orx neurons, Mch neurons are activated by glucose^96^ but are not activated by ghrelin^97^. Their activity increases in response to both discrete and contextual food-predictive cues, and this neural activity is closely linked with the behavioral response to these cues^13^. Interestingly, the activity of Mch neurons, unlike Orx neurons, does not diminish once food consumption begins; instead, their activity increases further during eating after the appetitive phase, suggesting that Mch neurons may sustain prolonged eating by reinforcing the positive feedback associated with nutrient intake^13,74,98^ (Fig. 3d).

## The role of LH in associative learning

The first phase of eating behavior, the appetitive phase, is thought to be primarily driven by the activity of Agrp neurons in the ARC^75,99,100^. These neurons are activated by energy deprivation and are inhibited before the consummatory phase by the sensory detection of food or food-predicting cues^78,79^. Optogenetic activation of Agrp neurons drives food seeking and increases food intake, while inhibition reduces food intake^99,100^. However, initiation of the appetitive phase and the transition from the appetitive phase to the consummatory phase in response to sensory information are not solely influenced by AgRP neurons. It is thought that LH plays a critical role either upstream or downstream of this process^41,60,75^.

First, the inhibition of Agrp neurons by food-predicting cues requires prior learning and memory that associate the cue with the food, a process supported by the LH. Signal that inhibits Agrp neurons by food-predicting cues is transmitted from LH glutamatergic neurons via DMH Lepr neurons^41^. LH glutamatergic neurons are rapidly activated by food-predicting cues, with the degree of activation proportional to the caloric value, and consequently trigger the rapid inhibition of Agrp neurons in response to food-predicting cues^12,41,78,79,101^. Furthermore, Orx neurons, which are considered part of the LH glutamatergic neurons, are selectively activated by learned food cues^15,23,102^. As the activation of Orx neurons increases food intake, it is believed that LH Orx neurons are involved in cue-potentiated eating^55^.

The LH receives signals from forebrain regions such as the mPFC, enabling the expression of context-appropriate behaviors, such as food seeking^103,104^. This activity of the LH is not merely a passive role of relaying signals from the cortex to Agrp neurons in the ARC, but is deeply involved in the organization of appetitive behavior through mediating associative learning between cues and food^41,105–107^. In the context of food seeking and the appetitive phase, in addition to LH glutamatergic neurons, LH GABAergic neurons are also essential for learning and expressing associations between food-predicting cues and rewards. Optogenetic inhibition studies reveal that LH GABAergic neurons are essential for learning food-predictive cue associations and expressing these learned associations^105^. Overall, a portion of LH neurons are a potential hub that facilitates associative learning between food and food-predicting cues and enhancing the incentive salience of food which optimizes and guides adaptive appetitive behavior^41,101,108^.

## The role of LH in different types of eating behavior

Eating behavior is regulated by two distinct systems: homeostatic and nonhomeostatic eating. Homeostatic eating maintains energy balance on the basis of physiological needs, whereas nonhomeostatic eating occurs independently of energy requirements, driven by external or emotional factors that may promote food consumption even when satiated.

### Homeostatic eating

Homeostatic eating is a physiological system that maintains energy balance by integrating peripheral metabolic signals with central neural circuits. The LH functions as a critical hub in this process, responding to circulating hormones such as leptin and ghrelin that signal energy status^50,109^. Within the LH, distinct neuronal populations coordinate this homeostatic response. Orx neurons regulate eating behaviors by influencing both appetite and metabolism^55^, while LepR neurons modulate eating behavior and energy expenditure through effects on locomotion and thermogenesis^48^. During hunger, Agrp neurons in the ARC project to the LH, driving sustained eating via positive reinforcement^60^. The LH integrates feedback signals through multiple mechanisms. Lepr neurons regulate eating behavior and energy expenditure^10,50^, exhibiting molecular adaptations to energy deficits^110^.

### Nonhomeostatic eating

Nonhomeostatic eating encompasses behaviors that occur independently of the body’s energy needs^2,5^. This type of eating can be triggered by external or emotional factors, leading to food consumption even in the absence of physiological hunger or after satiety is reached^5,38^. Nonhomeostatic eating can be categorized into distinct components, with pleasure-induced and stress-induced eating being two major categories that involve different LH circuits.

#### Pleasure-induced eating

Pleasure-induced eating is driven by the rewarding properties of food. The LH serves as a key node in reward circuits, utilizing its GABAergic and glutamatergic neuronal populations to process reward-related signals^12,43^. Activation of LH GABAergic neurons inhibits VTA GABAergic neurons, disinhibiting dopaminergic VTA neurons projecting to the NAc, thereby increasing dopamine levels^80^. The behavioral effects of LH GABAergic activation are evident in responses to palatable foods. Activation promotes the intake of palatable food and increases sucrose preference^2,38^. This selective response to palatable foods suggests that these neurons have a specialized role in pleasure-induced eating, distinct from the mechanisms governing homeostatic eating. While their inhibition reduces these behaviors^80^. The LH–VTA circuit appears particularly important in regulating pleasure-induced eating, as activating this pathway can elicit strong eating responses even in animals that are already sated. The role of the LH in pleasure-induced eating is further corroborated by electrophysiological evidence showing that these neurons encode the palatability of food rather than its caloric value^38^. Unlike neurons governing homeostatic eating, LH GABAergic neurons are primarily responsive to the rewarding properties of food, highlighting their specialized function in regulating pleasure-induced eating.

#### Stress-induced eating

Stress response within the LH involves specific circuits that regulate eating behavior under different stress conditions. When animals experience social stress, the synaptic strength between LHA and VTA glutamatergic neurons is enhanced, promoting increased consumption of palatable food^111^. The LH contains distinct neuronal populations that respond differently to stress. A specific population of LH penk neurons becomes activated by predator odor stress and drives high-fat diet overconsumption associated with negative emotional states^21^. Different types of stress activate distinct LH circuits to modulate eating behavior. The Nts neurons in LS projecting to the LH selectively activate during active escape situations and suppress eating behavior^18^. This provides a mechanism for switching from eating to defensive behaviors when threats are detected. By contrast, the LH GABAergic neurons to the DBB pathway help to suppress responses to anxiogenic environmental cues, promoting eating by reducing anxiety-like responses to environmental threats^53^.

## Future directions

The anatomical organization of the LH we present in this Review holds great promise in helping us understand the functional importance of different LH regions. Due to the lack of data available on the specific molecular identities of the LH anatomical regions, the organization we present here was based on stereotaxic coordinates provided by previous research on different cell types and circuits in the LH. We recognize the limitations of this method, and experimental techniques such as spatial transcriptomics should be used for a more accurate classification of cell types into the various anatomical regions of the LH.

The integrated temporal dynamics of LH circuits presented in this Review underscore the importance of fiber photometry data and precise single-cell event analyses with the use of microendoscopy techniques. The use of two-photon miniature microscope and electrode recording technologies promises greater temporal and spatial resolution, which would provide a clearer picture of how LH circuits interact in each phase of eating. Although we divided LH activity during eating behaviors into two phases, appetitive and consummatory, recent research demonstrates that distinct populations of neurons in the LH exhibit sequential firing patterns throughout the entire eating episode^112^. This suggests that the role of LH neurons may not be simply divided into appetitive and consummatory phases but involves neuronal populations that are sequentially activated across all eating phases, indicating that the appetitive and consummatory phases may be encompassed within the broader framework of sequential activation.

Increasing scientific interest in the LH has finally begun to deconstruct its profound anatomical and functional complexity, but much of the LH remains uncharted territory. While it has been revealed that distinct LH neurons are activated during appetitive and consummatory behaviors, pertinent data such as the molecular identity of each neuron, the activity of appetitive versus consummatory neurons time-locked to specific activity, and the projection sites of these neurons remain elusive. Applications of novel technologies such as Cal-Light (calcium and light-induced gene handling toolkit), which tags active neurons during specific activity^113^, or CaRMA (calcium and RNA multiplexed activity) imaging, which enables decoding the molecular identity of active neurons at a specific instant^114^, could greatly expedite this process and ultimately help in identifying promising targets for a host of new therapeutic interventions for eating-related disorders.

## Conclusion and clinical perspectives

The anatomically and functionally diverse LH neurons integrate various information from multiple brain regions and, therefore, serve as key regulators of eating behaviors. From a clinical perspective, bilateral implantation of LH deep brain stimulation electrodes has shown promise in treating patients with obesity^115,116^. In addition, we showed that viral delivery of chemogenetic modulators to LH GABAergic neurons can modulate eating behavior in nonhuman primates^4^. These recent findings underscore the urgent need for further research and hold great promise for opening new obesity therapeutic strategies, including drugs, devices and virally delivered drug-controlled gene therapy (chemogenetics).
